# Are consumption of dairy products and physical activity independently related to bone mineral density of 6-year-old children? Longitudinal and cross-sectional analyses in a birth cohort from Brazil

**DOI:** 10.1017/S1368980018001258

**Published:** 2018-05-16

**Authors:** Renata M Bielemann, Juliana dos S Vaz, Marlos R Domingues, Alicia Matijasevich, Iná S Santos, Ulf Ekelund, Bernardo L Horta

**Affiliations:** 1 Postgraduate Program in Epidemiology, Federal University of Pelotas, Rua Marechal Deodoro 1160 – 3º andar, Pelotas – RS, 96020-220, Brazil; 2 School of Nutrition, Federal University of Pelotas, Pelotas, RS, Brazil; 3 Postgraduate Program in Physical Education, Federal University of Pelotas, Pelotas, RS, Brazil; 4 Department of Preventive Medicine, School of Medicine, University of São Paulo, São Paulo, SP, Brazil; 5 Medical Research Council, Epidemiology Unit, University of Cambridge, Cambridge, UK; 6 Department of Sport Medicine, Norwegian School of Sport Sciences, Oslo, Norway

**Keywords:** Bone density, Exercise, Dairy products, Eating, Child

## Abstract

**Objective:**

To evaluate cross-sectional and longitudinal associations of consumption of dairy products and physical activity (PA) with bone mineral density (BMD).

**Design:**

Cohort study with children from the 2004 Pelotas (Brazil) Birth Cohort.

**Setting:**

Pelotas, a medium-sized Brazilian city.

**Subjects:**

The study started in 2004 and mothers/children were interviewed/measured periodically from birth to age 6 years. PA was measured by maternal proxy at 4 and 6 years and by accelerometry at 6 years. Consumption of dairy products was measured using 24 h food recall (at 4 years) and FFQ (at 6 years). Total-body and lumbar-spine BMD (g/cm^2^) were measured by dual-energy X-ray absorptiometry.

**Results:**

At 6 years, BMD was measured in 3444 children and 2636 children provided data on objectively measured PA by accelerometry. Consumption of dairy products at 4 years was associated with higher lumbar-spine BMD at 6 years in boys, while current consumption was positively associated with BMD in both sexes (*P* < 0·001). PA assessed by maternal report at 4 and 6 years of age was associated with higher BMD at 6 years in boys. PA assessed by accelerometry was positively related to total-body and lumbar-spine BMD in boys and lumbar-spine BMD in girls. We did not find evidence for an interaction between PA and consumption of dairy products on BMD.

**Conclusions:**

We observed positive and independent longitudinal and cross-sectional associations between consumption of dairy products and PA with BMD in the total body and at the lumbar spine in young children.

The physical capability across the lifespan is influenced by early factors that are related to the amount of ‘biological capital’ acquired during growing years. The peak of physical capability, as part of the ‘biological capital’, allows individuals to remain above a critical threshold of risk for adverse outcomes later in life^(^ ^1^ ^)^. Peak bone mass is one such example of the physical capability, partly influenced by early-life factors, contributing to reach the ‘full genetic potential’ which may then prevent osteoporosis-related fractures in the future^(^ ^2^ ^)^. The amount of nutrients available and the time spent in physical activity (PA), especially weight-bearing activities, are important in this context because the skeleton may not achieve its ‘full genetic potential’ if the supply of nutrients and/or mechanical loading during childhood and youth is insufficient^(^ ^2^ ^)^.

The importance of diet during childhood and adolescence to promote peak bone mass is well established and based on the role of dietary Ca^(^ ^2^ ^)^. Dairy products are probably the best dietary sources of Ca in childhood and dietary Ca is more strongly associated with bone mass compared with similar amounts provided by supplementation^(^ ^3^ ^)^. Thus, dairy products are considered an efficient source of nutrients for the bone because, besides Ca, they contain protein, Mg, K, Zn and P that are important nutrients in the construction of bone tissue^(^ ^4^ ^)^.

PA is an important modifiable factor influencing the development of bone mass. Previous randomized controlled trials reported greater bone mineral density (BMD) in children allocated to PA compared with controls^(^ ^5^ ^,^ ^6^ ^)^, confirmed by observational research^(^ ^7^ ^)^.

Although the positive relationship between dairy products, PA and BMD is recognized, some gaps remain. For example, it is unclear if the effects of Ca intake and PA on BMD are additive and/or interact. In addition, it is unclear if the response to PA and Ca intake on BMD is different among boys and girls before puberty^(^ ^5^ ^)^. Finally, there is no consensus on whether the positive effect of PA and Ca intake on BMD is cumulative during childhood, depending on how long children are exposed to higher levels of PA or Ca intake. Prospective examinations of the effect of PA and dietary factors, such as consumption of dairy products, on BMD among free-living young children are also scarce.

Thus, the aims of the present study were to: (i) assess the cross-sectional and longitudinal associations of consumption of dairy products and PA with BMD; (ii) examine the dose–response association of each individual exposure with the outcome; and (iii) evaluate if associations between exposures and BMD are additive or multiplicative. We used data from 4- and 6-year-old children belonging to the 2004 Pelotas (Brazil) Birth Cohort.

## Methods

Pelotas is a medium-sized city in Rio Grande do Sul (southern Brazil) with more than 300 000 inhabitants. In 2004, all maternity hospitals were visited daily from 1 January to 31 December and mothers were invited to participate, interviewed and their newborns were examined. All children and their mothers have been followed-up since then.

Follow-up assessments of the 2004 Pelotas Birth Cohort study were conducted during home visits at mean ages of 3·0 (sd 0·1), 11·9 (sd 0·2), 23·9 (sd 0·4) and 49·5 (sd 1·7) months and at the research clinic at 6·8 (sd 0·3) years. Detailed methods of the cohort are available elsewhere^(^ ^8^ ^)^.

Total-body and lumbar-spine (L1–L4) BMD (g/cm^2^) were measured by dual-energy X-ray absorptiometry (DXA; Lunar Prodigy Advance™, GE Healthcare, Germany). Data were not collected from disabled children or those presenting metal surgical implants and irremovable metal items. In total, 3444 children were scanned by DXA during the visit to the research clinic at mean age 6·8 years.

Interviews included a questionnaire to assess food consumption (at 4 and 6 years); a 24 h food recall (at 4 years) including milk, formula and yoghurt consumption; and an FFQ based on the 12 months prior to the interview at 6 years including whole and skimmed milk, yoghurt and cheese. Also, changes in consumption patterns from 4 to 6 years were evaluated based on the recommended daily ingestion of at least three portions of milk or dairy products according to the previous Brazilian Dietary Guidelines^(^ ^9^ ^)^.

PA was assessed by maternal report when children were 4 years old using the last question of the Netherlands Physical Activity Questionnaire (NPAQ)^(^ ^10^ ^)^. Mothers were asked to classify their children as ‘about equal’, ‘always’ or ‘almost always’ less or more physically active compared with other children of the same age. Scores ranged from 1 to 5 points from less to more active option. This question from NPAQ showed a correlation coefficient of 0·27 with daily minutes of moderate-to-vigorous PA (MVPA)^(^ ^10^ ^)^. Children were categorized into three PA groups according to maternal perception: ‘above average’ (4 or 5 points), ‘average’ (3 points) and ‘below average’ (1 or 2 points). We also considered the PA change from 4 to 6 years based on maternal perception using ‘above average’ as the category of reference.

PA was objectively measured by accelerometry in a sub-sample of children at 6·8 years (*n* 2636, 69 % of eligible children). The GENEActiv accelerometer is waterproof and measures acceleration in three axes (*x*, *y*, *z*) within a ±8 ***g*** dynamic range with a sampling frequency set at 85·7 Hz. Data are stored directly as sampled from the microelectromechanical systems chip (unfiltered) and expressed in units of m ***g*** (1000 m ***g*** = 1 ***g*** = 9·81 m/s^2^). The accelerometer was placed at the children’s non-dominant wrist and PA was assessed using a 24 h protocol for four to seven free-living days including at least one weekend day in all participants. Participants who visited the clinic on a Monday, Tuesday or Wednesday were monitored until the following Monday, whereas those who visited the clinic on a Thursday, Friday or Saturday were monitored until the following Wednesday. Following the free-living measurements, accelerometers were collected by the research team at the participants’ home. Children who were disabled or living in other cities were excluded from the measurements. More details on the collection of data from accelerometry are given in a previous publication^(^ ^11^ ^)^.

Accelerometry data were analysed with the GENEActiv software. Binary data were analysed with the R package GGIR (http://www.cran.r-project.org/web/packages/GGIR/vignettes/GGIR.html#citing-ggir). Detailed signal processing included verification of sensor calibration error using local gravity as reference, detection of sustained abnormally high values, non-wear detection, and exclusion of the first 10 h and last 20 h of the measurement. Calculation of the vector magnitude of activity-related acceleration was made using the Euclidian Norm minus 1 ***g*** (ENMO: 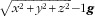 ) with any negative values rounded up to zero. Data were imputed for periods with invalid data and the average of similar time points on different days of the measurement was used. Valid data were present for every 15 min period in a 24 h cycle (even when scattered over multiple days). Procedures in the accelerometry analyses were conducted according to previous publications^(^ ^12^ ^–^ ^14^ ^)^.

Summary measurements were the average magnitude of wrist acceleration (overall volume, ENMO, m ***g***) and estimated time spent in 10 min bouts of MVPA. Daily time spent in MVPA was based on an intensity threshold of 100 m ***g*** (for each 5 s epoch data and 10 min bouts). Sensitivity analyses were also performed using time spent above a 200 m ***g*** cut-off point. Data from Hildebrand *et al*. suggest that 100 m ***g*** is similar to walking at 3 km/h in children and approximately 200 m ***g*** is equal to MVPA^(^ ^15^ ^)^. Data from accelerometry (acceleration in m ***g*** and MVPA in min/d) were analysed as continuous variables and categorized into quartiles.

The following variables were considered as potential confounders: child’s skin colour (white, black, brown or other); family income at birth (asked in the perinatal interview, being the sum of the earnings of the household members); maternal schooling at birth (in complete years of schooling); birth weight (measured by the hospital staff using paediatric scales (Filizola, São Paulo, Brazil) accurate to 10 g and calibrated weekly with standard weights; in grams); maternal smoking during pregnancy (asked in the perinatal interview; yes/no); maternal age at birth; breast-feeding duration (from information asked in all follow-up interviews up to 4 years of age; in months); and current height (measured to the nearest 1 mm, using a wooden stadiometer, in standing position).

All statistical analyses were performed with the statistical software package Stata version 12 and stratified by sex. Absolute and relative frequency of main exposures and confounders were described, as well as mean and standard deviation of both outcomes. Unadjusted and adjusted analyses were performed using linear regression and *P* values were obtained by Wald’s test for heterogeneity, using PA variables by proxy report and consumption of dairy products at both ages as ordinal variables (*β* coefficients calculated represent the difference in BMD for each category in relation to the reference group). Adjusted analyses included all confounders listed previously. Analyses did not include body weight as a confounder since it was not related to total-body or lumbar-spine BMD. Statistical procedures were performed using PA variables by proxy report and consumption of dairy products at both ages as ordinal variables and by classification according to status in exposures at both ages. To examine potential effect modification (in the present study, when the *P* values for the interaction term inserted in the analyses were <0·05), we included interaction terms (exposure×sex) for both exposures and also for the association of PA with BMD according to consumption of dairy products at each age (PA×consumption of dairy products at each age). The significance level was set at 5 %.

All follow-ups of the Pelotas 2004 Birth Cohort Study were approved by the Ethics Committee of the Federal University of Pelotas Medical School. All mothers signed an informed consent before any data collection.

## Results

In 2004, 4231 children were enrolled in the cohort. Between birth and follow-up at age 6 years, ninety-five children died. Of the original cohort, 3722 children were located and interviewed (follow-up rate=90·2 %). Of these, 3444 (92·5 %) children had valid data from the DXA scans whereas 2636 children provided at least two valid days of PA assessed by accelerometry (69·1 %). Children without data on objectively measured PA had higher total-body BMD than children with valid data (see online supplementary material, Supplemental Table 1). There was no other statistical difference between groups.

Table 1 shows the characteristics of boys and girls. Most mothers had at least 8 years of schooling and about 25 % were black or brown. More than 80 % and about 30 % of children reported consumption of cow’s milk and yoghurt at least once during the 24 h prior to the interview at 4 years of age, respectively. Approximately 30 % of boys and 20 % of girls reported cow’s milk consumption at least three times daily at 6 years of age, whereas most of the children (53 %) consumed dairy products, except for milk, at least once daily at age 6 years. Mothers classified approximately 50 % and 40 % of their children as ‘above average’ for PA at 4 and 6 years of age, respectively. More boys than girls were categorized into the two highest quartiles according to objectively measured PA. Total-body BMD at 6 years of age was on average greater in boys whereas lumbar-spine BMD was greater in girls.

**Table 1:** Sociodemographic characteristics, consumption of dairy products, physical activity (PA) and bone mineral density (BMD) in children belonging to the 2004 Pelotas (Brazil) Birth Cohort

	Boys		Girls	
	*n* or Mean	% or sd	*n* or Mean	% or sd
Maternal schooling (years)				
0–4 years	264	15·0	253	15·4
5–8 years	734	41·6	701	42·6
9–11 years	585	33·1	553	33·6
≥12 years	182	10·3	140	8·5
Skin colour				
White	1176	67·3	1118	67·7
Black	233	13·3	192	11·6
Brown	256	14·7	240	14·5
Other	82	4·7	102	6·2
Daily frequency of consumption of cow’s milk at 4 years				
<1	311	18·0	295	18·2
1–2	680	39·1	681	42·1
3	547	31·6	484	29·9
≥4	195	11·3	157	9·7
Daily frequency of consumption of yoghurt at 4 years				
<1	1112	64·1	1086	67·1
1	417	24·1	362	22·4
≥2	204	11·8	169	10·5
Daily frequency of consumption of cow’s milk at 6 years				
<1	424	26·0	443	28·7
1–2	715	43·7	777	50·2
3	447	27·4	293	18·9
≥4	48	2·9	34	2·2
Daily frequency of consumption of yoghurt and cheese at 6 years				
<1	742	45·5	725	47·0
1	447	27·5	445	28·9
≥2	439	27·0	372	24·1
Maternal perception of PA at 4 years				
Below average	94	5·4	68	4·2
Average	800	46·2	716	44·3
Above average	837	48·4	832	51·5
Maternal perception of PA at 6 years				
Below average	188	10·8	168	10·2
Average	845	48·4	808	49·1
Above average	712	40·8	671	40·7
Overall PA by accelerometer at 6 years (m ***g***)	64·0	17·4	54·8	13·5
MVPA by accelerometer at 6 years (min)	55·6	38·4	33·4	186·3
Total-body BMD (g/cm^2^)	0·834	0·048	0·819	0·049
Lumbar-spine (L1–L4) BMD (g/cm^2^)	0·646	0·079	0·667	0·088

MVPA, moderate-to-vigorous PA.

The association between consumption of dairy products, mothers’ perception of children’s PA and total-body BMD is displayed in Table 2. Higher consumption of dairy products at both ages was positively associated with higher total-body BMD at 6 years in boys and girls (*P* < 0·001). The magnitude of the association was greater in boys for consumption of dairy products at 6 years than in girls. Consuming dairy products at least once daily was positively associated with total-body BMD; however, the magnitude of the association was greater in those consuming dairy products three or more times daily. Boys classified by their mothers as ‘below average’ for PA at 4 and 6 years presented total-body BMD 0·014 (95 % CI −0·024, −0·005) g/cm^2^ and 0·017 (95 % CI −0·024, −0·009) g/cm^2^ lower, respectively, compared with those classified as ‘above average’ for PA. Girls classified by their mothers as ‘below average’ for PA at 6 years showed 0·009 (95 % CI −0·017, −0·001) g/cm^2^ lower total-body BMD than those classified as ‘above average’ for PA.

**Table 2:** Consumption of dairy products and physical activity (PA) in childhood in relation to total-body bone mineral density (BMD) at 6 years of age in children belonging to the 2004 Pelotas (Brazil) Birth Cohort

	Total-body BMD (g/cm^2^)									
	Boys					Girls				
		Crude		Adjusted			Crude		Adjusted	
	*n*	*β* coefficient	95 % CI	*β* coefficient	95 % CI	*n*	*β* coefficient	95 % CI	*β* coefficient	95 % CI
Daily frequency of dairy products at 4 years*		*P* < 0·001		*P* < 0·001			*P* < 0·001		*P* < 0·001	
<1	176	Ref.		Ref.		163	Ref.		Ref.	
1–2	583	0·019	0·011, 0·027	0·011	0·004, 0·019	614	0·025	0·016, 0·033	0·018	0·009, 0·026
3	529	0·030	0·022, 0·038	0·021	0·013, 0·028	489	0·030	0·022, 0·039	0·022	0·014, 0·030
≥4	445	0·031	0·023, 0·039	0·022	0·014, 0·030	351	0·033	0·025, 0·042	0·025	0·016, 0·034
Daily frequency of dairy products at 6 years*		*P* < 0·001		*P* < 0·001			*P* < 0·001		*P* < 0·001	
<1	223	Ref.		Ref.		257	Ref.		Ref.	
1–2	621	0·021	0·014, 0·029	0·014	0·007, 0·021	666	0·016	0·009, 0·023	0·007	0·001, 0·014
3	359	0·034	0·026, 0·042	0·024	0·016, 0·032	321	0·027	0·019, 0·035	0·017	0·010, 0·025
≥4	424	0·036	0·028, 0·043	0·025	0·018, 0·033	297	0·026	0·018, 0·034	0·019	0·012, 0·027
Maternal perception of PA at 4 years†		*P* = 0·020		*P* = 0·009			*P* = 0·045		*P* = 0·110	
Below average	94	−0·014	−0·025, −0·004	−0·014	−0·024, −0·005	68	−0·015	−0·027, −0·003	−0·011	−0·023, 0·000
Average	800	−0·001	−0·006, 0·003	−0·004	−0·008, 0·001	716	0·000	−0·005, 0·005	−0·003	−0·008, 0·002
Above average	837	Ref.		Ref.		832	Ref.		Ref.	
Maternal perception of PA at 6 years†		*P* = 0·024		*P* < 0·001			*P* = 0·144		*P* = 0·060	
Below average	188	−0·011	−0·018, −0·003	−0·017	−0·024, −0·009	168	−0·005	−0·014, 0·003	−0·009	−0·017, −0·001
Average	845	−0·002	−0·007, 0·002	−0·006	−0·011, −0·002	808	0·002	−0·002, 0·007	0·000	−0·005, 0·005
Above average	712	Ref.		Ref.		671	Ref.		Ref.	

Ref., reference category. Adjusted for skin colour, family income at birth, maternal schooling, birth weight, maternal smoking during the pregnancy, maternal age at birth, breast-feeding duration and current height.

* Adjusted for current PA.

† Adjusted for current consumption of dairy products.

The consumption of dairy products three or more times daily at 4 years of age was positively associated with higher lumbar-spine BMD at 6 years in boys (Table 3). The daily frequency of consumption of dairy products at 6 years was positively associated with lumbar-spine BMD in both sexes. Consuming dairy products at least once daily at age 6 years was positively associated with lumbar-spine BMD in boys, whereas in girls the positive association was observed only in those who consumed dairy products three times daily. Boys classified as ‘below average’ for PA had on average 0·029 (95 % CI −0·045, −0·014) g/cm^2^ and 0·025 (95 % CI −0·037, −0·013) g/cm^2^ lower lumbar-spine BMD at 6 years than boys classified as ‘above average’ for PA at 4 and 6 years of age, respectively. Boys classified as ‘average’ for PA at both ages also had lower lumbar-spine BMD compared with more active boys. No association was found between maternal perception of PA and lumbar-spine BMD in girls at age 6 years.

**Table 3:** Consumption of dairy products and physical activity (PA) in childhood in relation to lumbar-spine (L1–L4) bone mineral density (BMD) at 6 years of age in children belonging to the 2004 Pelotas (Brazil) Birth Cohort

	Lumbar-spine BMD (g/cm^2^)									
	Boys					Girls				
		Crude		Adjusted			Crude		Adjusted	
	*n*	*β* coefficient	95 % CI	*β* coefficient	95 % CI	*n*	*β* coefficient	95 % CI	*β* coefficient	95 % CI
Daily frequency of dairy products at 4 years*		*P* < 0·001		*P* < 0·001			*P =* 0·003		*P* = 0·398	
<1	176	Ref.		Ref.		160	Ref.		Ref.	
1–2	577	0·019	0·005, 0·032	0·006	−0·006, 0·019	606	0·028	0·012, 0·044	0·012	−0·002, 0·027
3	525	0·029	0·015, 0·042	0·014	0·001, 0·026	480	0·026	0·010, 0·042	0·011	−0·004, 0·026
≥4	445	0·036	0·022, 0·051	0·023	0·010, 0·036	347	0·029	0·012, 0·046	0·012	−0·003, 0·028
Daily frequency of dairy products at 6 years*		*P* < 0·001		*P* = 0·009			*P* < 0·001		*P* = 0·031	
<1	222	Ref.		Ref.		252	Ref.		Ref.	
1–2	616	0·026	0·014, 0·038	0·012	0·001, 0·023	655	0·024	0·011, 0·037	0·010	−0·002, 0·022
3	358	0·040	0·027, 0·053	0·021	0·009, 0·033	318	0·033	0·019, 0·045	0·021	0·007, 0·035
≥4	418	0·035	0·023, 0·048	0·016	0·004, 0·028	292	0·022	0·007, 0·037	0·013	−0·001, 0·027
Maternal perception of PA at 4 years†		*P* < 0·001		*P* < 0·001			*P* = 0·120		*P* = 0·167	
Below average	94	−0·033	−0·050, −0·016	−0·029	−0·045, −0·014	66	−0·024	−0·046, −0·001	−0·020	−0·041, 0·001
Average	795	−0·007	−0·015, 0·001	−0·010	−0·018, −0·003	706	−0·001	−0·010, 0·008	−0·003	−0·011, 0·006
Above average	832	Ref.		Ref.		820	Ref.		Ref.	
Maternal perception of PA at 6 years†		*P* = 0·019		*P* < 0·001			*P* = 0·419		*P* = 0·163	
Below average	188	−0·018	−0·031, −0·005	−0·025	−0·037, −0·013	169	−0·009	−0·025, 0·006	−0·013	−0·027, 0·001
Average	838	−0·006	−0·014, 0·002	−0·011	−0·018, −0·003	795	0·000	−0·009, 0·010	0·000	−0·009, 0·008
Above average	707	Ref.		Ref.		658	Ref.		Ref.	

Ref., reference category. Adjusted for skin colour, family income at birth, maternal schooling, birth weight, maternal smoking during the pregnancy, maternal age at birth, breast-feeding duration and current height.

* Adjusted for current PA.

† Adjusted for current consumption of dairy products.

The association between objectively measured PA and BMD is shown in Table 4. Both overall PA (*P* = 0·018 for total-body BMD; *P* = 0·002 for lumbar-spine BMD) and time spent in MVPA (*P* = 0·009 for total-body BMD; *P* = 0·024 for lumbar-spine BMD) were positively associated with total-body and lumbar-spine BMD in boys. In girls, overall PA and time spent in MVPA were positively associated with lumbar-spine BMD (*P* = 0·002 and *P* = 0·029, respectively). Results using information on objectively measured PA with the 200 m ***g*** cut-off point were not statistically associated with BMD (see online supplementary material, Supplemental Table 2).

**Table 4:** Association between objectively measured (accelerometer) physical activity (PA) and bone mineral density (BMD) at 6 years of age in children belonging to the 2004 Pelotas (Brazil) Birth Cohort

	BMD (g/cm^2^)									
	Boys					Girls				
		Crude		Adjusted			Crude		Adjusted	
	*n*	*β* coefficient	95 % CI	*β* coefficient	95 % CI	*n*	*β* coefficient	95 % CI	*β* coefficient	95 % CI
Total-body BMD (g/cm^2^)										
Overall PA (m ***g***)	1277	0·0000	−0·0002, 0·0001	0·0002	0·0001, 0·0004	1214	−0·0001	−0·0003, 0·0001	0·0002	0·0000, 0·0003
MVPA (min)	1280	0·0000	0·0000, 0·0001	0·0001	0·0000, 0·0002	1214	0·0000	−0·0001, 0·0001	0·0001	0·0000, 0·0002
Lumbar-spine BMD (g/cm^2^)										
Overall PA (m ***g***)	1269	−0·0001	−0·0003, 0·0002	0·0004	0·0002, 0·0006	1194	−0·0001	−0·0005, 0·0003	0·0006	0·0002, 0·0009
MVPA (min)	1272	0·0000	−0·0001, 0·0001	0·0001	0·0000, 0·0002	1194	−0·0001	−0·0002, 0·0001	0·0002	0·0000, 0·0004

MVPA, moderate-to-vigorous PA. Adjusted for skin colour, family income at birth, maternal schooling, birth weight, maternal smoking during the pregnancy, maternal age at birth, breast-feeding duration, current height and current consumption of dairy products.

**Fig. 1:**
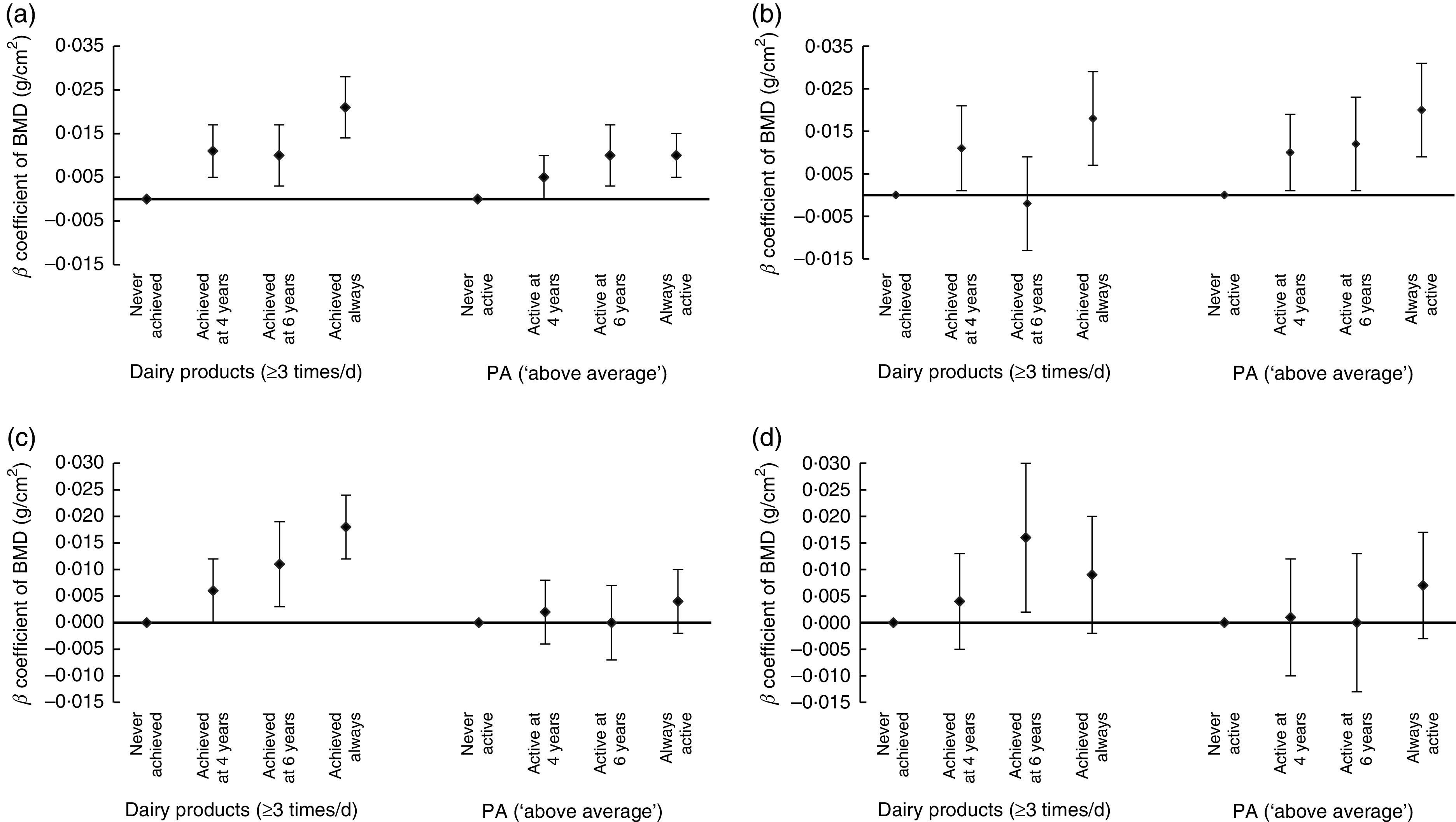
Association of variation in adequate consumption of dairy products and physical activity (PA) during childhood with bone mineral density (BMD) of children from the 2004 Pelotas (Brazil) Birth Cohort at 6 years of age: (a) total-body BMD in boys; (b) lumbar-spine (L1–L4) BMD in boys; (c) total-body BMD in girls; (d) lumbar-spine (L1–L4) BMD in girls. Values are *β* coefficients with their 95 % CI represented by vertical bars, with ‘never achieved’ and ‘never active’ as reference categories, adjusted for skin colour, family income at birth, maternal schooling, birth weight, maternal smoking during the pregnancy, maternal age at birth, breast-feeding duration and current height

Figure 1 shows that achieving the recommended levels of dairy product intake (≥3 portions of milk or dairy products daily) at both 4 and 6 years of age was positively associated with total-body BMD at 6 years of age in boys (*P* < 0·001). Girls who adhered to the recommendation for consumption of dairy products at age 6 years or at both time points had greater total-body BMD at age 6 years than girls who never adhered to the recommendation (*P*<0·001). For lumbar-spine BMD at 6 years, boys who reached the recommended consumption of dairy products at 4 years or at both time points had greater BMD than boys who never adhered to the recommendation (*P*<0·001). In girls, the positive association was observed only when there was adequate consumption at 6 years (*β* = 0·016; 95 % CI 0·002, 0·030).

Boys classified by their mothers as ‘above average’ for PA only at 6 years or at both ages had greater total-body BMD at 6 years than boys never classified as ‘above average’ (*P* = 0·002). For lumbar-spine BMD, boys classified as ‘above average’ for PA at any age had greater BMD than boys never classified as ‘above average’, although a higher *β* coefficient was found in always above average boys (*P*<0·001). Variation on PA (maternal perception) was not related to either total-body or lumbar-spine BMD in girls (*P* = 0·472 and *P* = 0·578, respectively).

Results presented are stratified by sex, although tests for effect modification of each exposure on outcomes according to sex showed no statistical significance for most exposures except for variation in adequate consumption of dairy products from 4 to 6 years (*P* = 0·021) with lumbar-spine BMD.

When the relationship between PA and BMD was stratified according to adequacy of consumption of dairy products, we observed a positive association of being classified as ‘above average’ for PA at 4 years on BMD with consumption of dairy products lower than three times daily in girls (total-body BMD, *β* = 0·021; 95 % CI 0·006, 0·036; lumbar-spine BMD, *β* = 0·039; 95 % CI 0·014, 0·064). On the other hand, the same analysis indicated that boys who were ‘average’ or ‘above average’ for PA at 4 and 6 years had greater lumbar-spine BMD only if consumption of dairy products was adequate (PA at 4 years: ‘average’, *β* = 0·029; 95 % CI 0·006, 0·052; ‘above average’, *β* = 0·042; 95 % CI 0·020, 0·065; PA at 6 years: ‘average’, *β* = 0·025; 95 % CI 0·008, 0·041; ‘above average’, *β* = 0·036; 95 % CI 0·019, 0·053). However, when using objectively measured PA, a positive association was observed between PA and lumbar-spine BMD in those boys who reported an inadequate consumption of dairy products (*β* = 0·0006; 95 % CI 0·0002, 0·0009). This was not observed among girls.

## Discussion

The present study assessed the cross-sectional and longitudinal associations between PA and consumption of dairy products and BMD among children from a Southern Brazilian birth cohort. To our knowledge, it is the first observational prospective study to report these associations in children from Latin America. The consumption of dairy products and PA seem to be equally important to BMD in childhood. Associations of greater magnitude were found for total-body BMD in comparison to those observed for lumbar-spine BMD in the relationship with daily frequency of consumption of dairy products, whereas PA coefficients were greater in magnitude in relation to lumbar-spine BMD. Consumption of dairy products and PA at 4 years of age were positively related to BMD in both anatomical sites, with similar results at 6 years. Maintenance of adequate consumption of dairy products and high PA in a 2-year period was beneficial to BMD at both sites in boys. Results seemed to be more consistent among boys than girls although statistical tests for interaction were significant only for consumption of dairy products, possibly indicating a sex difference already from childhood that may manifest in substantial sex differences by age.

The Dietary Reference Intakes established by the Institute of Medicine^(^ ^16^ ^)^ recommend a daily Ca intake of 800 mg/d for children aged 4–8 years. This recommendation is exemplified as a daily consumption of 480 ml of milk or two or three portions of dairy products^(^ ^9^ ^,^ ^17^ ^)^. The premise of this recommendation is that milk during childhood has a positive impact on current and future bone health^(^ ^18^ ^)^. Maintenance of adequate Ca intake at young ages positively predicted BMD status in prepubertal and postpubertal years^(^ ^19^ ^)^. Daily consumption of two or more servings of dairy products starting at 3–5 years of age was positively associated with higher BMD and bone area in adolescents aged 15–17 years^(^ ^20^ ^)^. Besides, the positive effect of dairy consumption on bone mass accretion or density in childhood is also supported by 1- to 2-year randomized trials supplementing dairy foods or milk extract in prepubertal children with low baseline Ca intake^(^ ^21^ ^,^ ^22^ ^)^.

We observed a decrease in the frequency of milk consumption followed by an increase in yoghurt and cheese consumption from 4 to 6 years old (Table 1). This finding corroborates a previous study that reported a positive time trend in cheese and yoghurt consumption compensated for the decrease in milk during childhood^(^ ^23^ ^)^. Although the consumption of dairy products seems more beneficial for bone mass accretion than Ca supplements^(^ ^3^ ^)^, children who decrease daily milk consumption less frequently meet the total Ca recommendation, which may indicate replacement by smaller dairy portion sizes or milk-based beverages with lower Ca content^(^ ^19^ ^)^.

Our study provided evidence of a positive association between consumption of dairy products and total-body and lumbar-spine BMD at early age. Daily consumption of dairy products during childhood is also reported as a positive dietary factor associated with greater BMD in adulthood^(^ ^24^ ^)^, but evidence is still inconsistent^(^ ^25^ ^,^ ^26^ ^)^. When children’s diet is supplied with Ca through dietary products, studies report a gain in total-body BMD, especially at the lumbar spine and hip^(^ ^21^ ^,^ ^27^ ^,^ ^28^ ^)^. By contrast, long-term deprivation of milk in childhood is suggested as a risk factor for smaller skeleton size and significantly lower bone area, total-body and site-specific BMD^(^ ^29^ ^,^ ^30^ ^)^, although lower bone area may be a consequence of small body size.

Several studies have shown the beneficial effect of PA in pre- and postpubertal childhood on bone mass^(^ ^31^ ^–^ ^34^ ^)^. Results from prospective studies in young children are scarce but cross-sectional analyses suggest positive associations between PA and BMD, suggesting a short-term benefit^(^ ^35^ ^,^ ^36^ ^)^. Our observations extend these observations, including a 2-year follow-up period.

Although PA in young adulthood is positively related to an increase in adult BMD^(^ ^37^ ^,^ ^38^ ^)^, some authors describe the growth period (childhood and adolescence) as the best opportunity to improve bone mass^(^ ^39^ ^–^ ^42^ ^)^. However, there is no consensus on whether the effect of PA is most prominent before or after puberty although some have suggested that PA during the most active period of maturity plays an important role in optimizing bone mass^(^ ^41^ ^,^ ^42^ ^)^. On the other hand, it has also been suggested that the prepubertal years, in which the presence of growth hormone is more expressive than of sex steroids, is a sensitive period to increase BMD^(^ ^43^ ^)^. Future follow-up of the current cohort throughout puberty may resolve these issues.

PA is especially important to promote increases in BMD due to mechanical loading. Different mechanisms are related to increase in BMD during puberty in boys and girls, whereas an increase in periosteal apposition in both sexes is suggested to be the main mechanism related to increase in BMD due to mechanical loading prior to puberty^(^ ^44^ ^)^. This may explain the lack of statistical interaction between PA and sex in association with BMD in the current cohort. The magnitude of associations between PA and BMD was greater in boys than in girls based on maternal proxy report. However, even if boys and girls are classified in the same category by maternal proxy report, the higher overall PA (64·0 *v*. 54·8 m ***g***) and time spent in MVPA (55·6 *v*. 33·3 min) assessed by accelerometry at age 6.8 years observed in boys indicate differences in objectively measured PA for the same maternal perception of PA. This may potentially contribute to the difference in the magnitude of association between sexes.

Previous randomized controlled trials and studies in adults have shown that the magnitude of the association between PA and BMD is greater for weight-bearing sites, such as the lumbar spine and femoral neck^(^ ^5^ ^,^ ^38^ ^,^ ^45^ ^)^. These sites are more susceptible to bone adaptation promoted by loading induced by weight-bearing PA^(^ ^45^ ^)^. Our findings are in line with the literature since greater magnitudes of association for both longitudinal and cross-sectional analyses between maternally reported PA and objectively measured PA with BMD were observed for the lumbar spine site than for total-body BMD. In contrast, the magnitude of association was similar between the two anatomical sites for consumption of dairy products, suggesting a similar association between accrual and growth of the bone at both anatomical sites and the consumption of dairy products.

Our results on an association between consumption of dairy products and PA and BMD in children from Latin America are novel. This is because BMD is influenced by ethnicity^(^ ^46^ ^,^ ^47^ ^)^ and possibly also by socio-economic status^(^ ^48^ ^,^ ^49^ ^)^, factors that may differ substantially between low- or middle-income countries and high-income countries, in which previous studies have been conducted. Further, types and amounts of dairy products consumed and PA levels may also differ between low- or middle-income countries and high-income countries, as previously observed^(^ ^50^ ^)^.

Some limitations should be acknowledged when interpreting our observations. First is the use of different methods to estimate the consumption of dairy products at 4 and 6 years of age. The use of only one 24 h dietary recall is a common practice in population-based studies, although the use of multiple 24 h dietary recalls increases the accuracy of the method^(^ ^51^ ^)^. However, a previous study examined the first three components defined by principal component analysis from a 24 h food recall for children and found that the results were very similar to those obtained using the FFQ^(^ ^52^ ^)^. In addition, as a monotonous diet was previously reported in Brazilian children^(^ ^53^ ^,^ ^54^ ^)^, the use of one single previous 24 h food recall likely reflects the food habits of the children. Other limitations are the absence of information on BMD at age 4 years, limiting longitudinal inferences. Not all children provided valid data on objectively measured PA, which may influence the results. However, our study is still one of the largest to date combining objectively measured PA with data on BMD from DXA scans in young children. Finally, we cannot exclude the possibility that our results are explained by unmeasured (e.g. genotype) or poorly measured confounders.

The use of maternal perception of her child’s PA may also influence our results. Measuring self-reported PA in children is a challenge and the use of accelerometers is preferable. However, due to logistical and cost reasons, objective measures were available only at 6 years of age. On the other hand, self-reported PA was positively associated with accelerometry in a previous study carried out with children from the same city, with correlation coefficients similar to those found for other self-reported instruments used at different ages^(^ ^10^ ^)^, although the use of this subjective method is an important limitation. Furthermore, an increase in acceleration and time spent in MVPA was positively related to mothers’ perception of PA, mainly in boys (data not shown). This reduces the risk of bias due to self-reported PA, although accelerometry does not provide information on the kind of PA performed.

Results from accelerometers showed two interesting findings: overall amount of PA was favourably related to BMD; however, bouts of at least moderate-intensity may also contribute to enhanced BMD, whereas accumulating MVPA in shorter epochs appears unrelated to BMD. Further studies are warranted to confirm these observations.

As previously described in the ‘Methods’, body weight was not included in the adjusted analysis since it was not related to total-body BMD (*P* = 0·178 in boys; *P* = 0·610 in girls) or lumbar-spine BMD (*P* = 0·397 in boys; *P* = 0·700 in girls). In addition, even in the case of an association, since body weight is influenced by energy expenditure from PA and by food intake, body weight could be a mediating variable in the analyses. Thus, body weight did not meet the criteria to be considered a possible confounder in our analyses.

## Conclusion

In conclusion, consumption of dairy products was positively associated with BMD in the total body and at the lumbar spine in young Brazilian children. PA assessed by maternal proxy report (only in boys) and objectively measured by accelerometry (overall PA and time spent in MVPA using 10 min bouts) was also positively associated with BMD, particularly at the lumbar spine site. These findings support the evidence of a cumulative effect of PA and consumption of dairy products on bone mineral accrual during growth.

## Supplementary material

For supplementary material accompanying this paper visit https://doi.org/10.1017/S1368980018000940. click here to view supplementary material
