# Supplementary material for: Are consumption of dairy products and physical activity independently related to bone mineral density of 6-year-old children? Longitudinal and cross-sectional analyses in a birth cohort from Brazil
Source: Public Health Nutr. 2018 May 16;21(14):2654–64. doi: 10.1017/S1368980018001258 (PMC6141993; doi:10.1017/S1368980018001258)
Supplement: Supplementary file 1 [file S1368980018001258sup001.docx]

**Supplementary table 1.** Sociodemographic characteristics, consumption of dairy products, physical activity and bone density among children belonging to the 2004 Pelotas (Brazil) Birth Cohort.

| **Variables** | **Children with no information from Acc (%)** | **Children with information from Acc (%)** | **p** |
| --- | --- | --- | --- |
| **Sex** |  |  | 0.241 |
| Boys | 723 (50.7) | 1183 (52.6) |  |
| Girls | 704 (49.3) | 1064 (47.4) |  |
| **Maternal Schooling** (years) |  |  | 0.172 |
| 0 – 4 years | 232 (16.7) | 336 (15.1) |  |
| 5 – 8 years | 550 (39.5) | 943 (42.4) |  |
| 9 – 11 years | 457 (32.9) | 735 (33.1) |  |
| **≥** 12 years | 151 (10.9) | 208 (9.4) |  |
| **Skin Color** |  |  | 0.614 |
| White | 634 (68.1) | 1494 (67.2) |  |
| Non-white | 297 (31.9) | 730 (32.8) |  |
| **Daily frequency of consumption of cow’s milk at 4 years** |  |  | 0.845 |
| < 1 | 202 (18.9) | 397 (18.1) |  |
| 1 – 2 | 426 (39.9) | 910 (41.5) |  |
| 3 | 329 (30.8) | 661 (30.1) |  |
| ≥ 4 | 111 (10.4) | 226 (10.3) |  |
| **Daily frequency of consumption of yogurt at 4 years** |  |  | 0.722 |
| < 1 | 710 (66.4) | 1430 (65.2) |  |
| 1 | 241 (22.6) | 522 (23.8) |  |
| ≥ 2 | 117 (11.0) | 242 (11.0) |  |
| **Daily frequency of consumption of cow’s milk at 6 years** |  |  | 0.459 |
| < 1 | 235 (27.3) | 570 (27.4) |  |
| 1 – 2 | 423 (49.0) | 965 (46.4) |  |
| 3 | 182 (21.1) | 491 (23.6) |  |
| ≥ 4 | 22 (2.6) | 54 (2.6) |  |
| **Daily frequency of consumption of yogurt and cheese at 6 years** |  |  | 0.623 |
| < 1 | 388 (45.1) | 965 (46.6) |  |
| 1 | 252 (29.3) | 571 (27.6) |  |
| ≥ 2 | 220 (25.6) | 534 (25.8) |  |
| **Physical activity at 4 years** |  |  | 0.221 |
| Below average | 65 (6.1) | 106 (4.9) |  |
| Average | 463 (43.4) | 998 (45.5) |  |
| Above average | 540 (50.5) | 1088 (49.6) |  |
| **Physical activity at 6 years** |  |  | 0.165 |
| Below average | 116 (12.5) | 226 (10.2) |  |
| Average | 376 (40.4) | 909 (41.0) |  |
| Above average | 438 (47.1) | 1083 (48.8) |  |
| **Total body BMD** (g/cm^2^) [mean (sd)] | 0.830 (0.002) | 0.825 (0.001) | 0.013 |
| **Lumbar spine BMD** (g/cm^2^) [mean (sd)] | 0.658 (0.003) | 0.656 (0.001) | 0.484 |

Acc – Accelerometry; MVPA – Moderate-to-vigorous physical activity; BMD – Bone mineral density.

**Supplementary table 2.** Association between time spent in objectively measured acceleration ≥ 200 m*g* and bone mineral density at 6 years of age in children belonging to the 2004 Pelotas Birth Cohort.

|  | **Bone Mineral Density (g/cm^2^)** | | | | | |
| --- | --- | --- | --- | --- | --- | --- |
|  | **Boys** | | | **Girls** | | |
|  | **n** | **Crude** | **Adjusted** | **n** | **Crude** | **Adjusted** |
|  |  | **β coefficient (95%CI)** | **β coefficient (95%CI)** |  | **β coefficient (95%CI)** | **β coefficient (95%CI)** |
| **Total Body BMD (g/cm^2^)** |  |  |  |  |  |  |
| **Acceleration ≥ 200 m*g*** (continuous) | 1092 | p=0.311  0.0000 (-0.0001; 0.0000) | p=0.731  0.0000 (-0.0001; 0.0001) | 1031 | p=0.956  0.0000 (-0.0001; 0.0001) | p=0.639  0.0000 (-0.0001; 0.0001) |
| **Acceleration ≥ 200 m*g*** | 1092 | p=0.835 | p=0.381 | 1031 | p=0.948 | p=0.860 |
| 1^st^ (lowest) |  | Ref. | Ref. |  | Ref. | Ref. |
| 2^nd^ |  | 0.0020 (-0.0062; 0.0101) | 0.0009 (-0.0070; 0.088) |  | 0.0008 (-0.0078; 0.0093) | 0.035 (-0.0045; 0.0115) |
| 3^rd^ |  | -0.0014 (-0.0095; 0.0067) | -0.0049 (-0.127; 0.0030) |  | 0.0024 (-0.0060; 0.0109) | 0.0020 (-0.0058; 0.0098) |
| 4^th^ (highest) |  | -0.0011 (-0.0094; 0.0072) | 0.0009 (-0.0072; 0.0090) |  | 0.0005 (-0.0078; 0.0088) | 0.0013 (-0.0065; 0.0091) |
| **Lumbar Spine BMD (g/cm^2^)** |  |  |  |  |  |  |
| **Acceleration ≥ 200 mg** (continuous) | 1088 | p=0.325  -0.0001 (-0.0002; 0.0001) | p=0.858  0.0000 (-0.0001; 0.0001) | 1014 | p=0.861  0.0000 (-0.0002; 0.002) | p=0.388  0.0001 (-0.0001; 0.0002) |
| **Acceleration ≥ 200 m*g*** | 1088 | p=0.359 | p=0.426 | 1014 | p=0.869 | p=0.710 |
| 1^st^ (lowest) |  | Ref. | Ref. |  | Ref. | Ref. |
| 2^nd^ |  | 0.0101 (-0.0035; 0.0237) | 0.0083 (-0.0039; 0.0206) |  | 0.0036 (-0.0126; 0.0198) | 0.0058 (-0.0094; 0.0210) |
| 3^rd^ |  | 0.0004 (-0.0131; 0.0140) | -0.0007 (-0.0130; 0.0115) |  | 0.0069 (-0.0091; 0.0229) | 0.0084 (-0.0064; 0.0232) |
| 4^th^ (highest) |  | 0.0000 (-0.0139; 0.0140) | 0.0032 (-0.0094; 0.0158) |  | 0.0034 (-0.0124; 0.0192) | 0.0065 (-0.0083; 0.0213) |

Adjusted for skin color, family income at birth, maternal schooling, birth weight, maternal smoking during the pregnancy, maternal age at birth, breastfeeding duration, current height and current consumption of dairy products.

Data are b-coefficients and 95% confidence intervals.
